# Prevalence and related factors of abdominal obesity among urban adults aged 35 to 79 years in southwest China

**DOI:** 10.3389/fpubh.2023.1117897

**Published:** 2023-11-08

**Authors:** Chuan Huang, Ying Zhang, Ya Liu, Jian-Xiong Liu, Yong-Mei Hu, Wei-Wei Tang, Tzung-Dau Wang, Xiao-bo Huang

**Affiliations:** ^1^Department of Cardiology, The Second People’s Hospital of Chengdu, Chengdu, Sichuan, China; ^2^Department of Neurology, The Second People’s Hospital of Chengdu, Chengdu, Sichuan, China; ^3^Department of Endocrinology and Metabolism, Second People’s Hospital of Chengdu, Chengdu, Sichuan, China; ^4^School of Health Policy and Management, Nanjing Medical University, Nanjing, Jiangsu, China; ^5^Jiangsu Provincial Institute of Health, Nanjing Medical University, Nanjing, Jiangsu, China; ^6^Center for Global Health, Nanjing Medical University, Nanjing, Jiangsu, China; ^7^Department of Internal Medicine, Cardiovascular Center and Division of Cardiology, National Taiwan University Hospital, Taipei City, Taiwan, China

**Keywords:** prevalence, related factor, abdominal obesity, urban adults, southwest China

## Abstract

**Objectives:**

This study aimed to investigate the prevalence and related factors of abdominal obesity among urban adults aged 35 to 79 years in southwest China.

**Methods:**

From September 2013 to March 2014, a multi-stage sampling was conducted, and a total of 10,981 people aged 35–79 years living in Chengdu and Chongqing were included. More than 30 investigators were trained in data collection, including questionnaire, anthropometric measurements and blood biomarkers testing. Abdominal obesity was defined as waist circumference ≥ 90 cm for men and ≥ 85 cm for women.

**Results:**

The prevalence of abdominal obesity was 30.7%, 24.8% in males and 33.9% in females (*p* < 0.001). The prevalence of abdominal obesity increased with BMI. The prevalence of abdominal obesity was positively correlated with age, sex, marriage, alcohol consumption, hypertension and diabetes, and negatively correlated with high education level, smoking and Physical activity.

**Conclusion:**

The prevalence of abdominal obesity among adults aged 35–79 in urban communities in southwest China is high, which is close to that of adults in urban communities in China. We should strengthen health education among the population, adopt healthy diet, maintain moderate physical activity and other measures to curb the prevalence of abdominal obesity in urban communities in southwest China.

## Introduction

Overweight and obesity are important risk factors for coronary heart disease, diabetes, stroke and chronic kidney disease (1–3). About 4 million deaths worldwide are directly attributed to high BMI, which accounts for 7.1 percent of all deaths (4). With the rapid development of China’s economy and society, the prevalence of overweight and obesity is rising (5, 6). In southwest China, 200 million people live in an area of more than 2.3 million square kilometers, accounting for one quarter of China’s land area (7). The implementation of China’s western development strategy since 2000 has led to significant economic and infrastructure growth in southwest China. At the same time, the prevalence of overweight and obesity is also increasing, which may change with economic development and lifestyle changes. Current obesity epidemic data are based on BMI analysis. While BMI is the most commonly used tool for measuring obesity, it mainly reflects body fat, not visceral fat (8–10). Previous research has shown that abdominal obesity is a key factor in cardiovascular disease risk (9, 10). In order to effectively control the obesity epidemic, it is necessary to understand the prevalence of abdominal obesity. However, large-scale epidemiological investigations of abdominal obesity have been lacking in the region. Therefore, from 2013 to 2014, we conducted an epidemiological investigation of abdominal obesity in Chengdu and Chongqing and analyzed the related factors, which provided a certain theoretical basis for the prevention and treatment of obesity and cardiovascular and cerebrovascular diseases in this region.

## Methods

### Study population

From September 2013 to March 2014, a cross-sectional survey was conducted among urban community population over 35 years old in Chengdu and Chongqing by means of multi-stage stratified sampling. The first step was to randomly sample Jinjiang District, Longquanyi District and Chenghua District as the urban areas of Chengdu, Yubei district and Jiangbei District as the urban areas of Chongqing. The second step was to randomly sample a subdistrict from each district. In the third step, a community was randomly sampled from each subdistrict, and a total of 5 communities were sampled. In each community according to the inclusion and exclusion criteria of this study, the survey team selected community residents with buildings as the basic unit, registered a total of 14,061 eligible participants, and finally 13,378 adults aged 35 to 79 were included in the survey. Due to the lack of demographic information and blood pressure, weight, lipid measurements, or waist circumference, and body mass index (BMI) data, 10,981 participants were included in the final analysis.

In 2004, Data from CCDRFS indicate that the prevalence of abdominal obesity among adults in China was 25.9%, to achieve a margin of error (ε) of 2%, so, according to the sample size calculate formula, which would require the sample size to reach 1844. Giving a 10% missing rate, it would require us to survey 2029 individuals for each community. Total 5 communities from Chengdu and Chongqing, so the sample size has least to reach 10,145.

This study has been reviewed by the Ethics Committee of Chengdu Second People’s Hospital (no 2013015). All respondents signed written informed consent forms.

### Inclusion and exclusion criteria

Adults between the ages of 35 and 79 who had lived in selected communities for more than five years were included in the study from September 2013 to March 2014. People with malignancy, mental illness, secondary hypertension, kidney failure requiring dialysis, or refusal to participate were excluded.

### Data collection

More than 30 medical staffs had been uniformly trained and qualified before taking up their works. The project team carried out field investigation with a unified questionnaire design. The questionnaire included general demographic information, lifestyle, personal and family history (family history of overweight and obesity), height, weight and other physical examinations. Measurement of height and weight requires participants to take off shoes, hat and trousers. Body mass index (BMI) = weight/height (2) (Kg/m^2^). Waist circumference (WC) was measured as the minimum circumference between the inferior margin of the ribcage and the iliac crest (11). The blood pressure was measured with an Omron arm electronic sphygmomanometer after 15 min rest. The blood pressure of the right upper arm was measured twice in the sitting position, and the mean value was taken. Blood sample collection and laboratory examination: after fasting for at least 12 h, fasting venous blood of patients from morning onwards and 75 g glucose 2 h postprandial venous blood were extracted, and blood glucose, lipid and uric acid were detected. Total cholesterol (TC), triglycerides (TG) and blood glucose were measured by oxidase method. The levels of high-density lipoprotein cholesterol (HDL-C) and low-density lipoprotein cholesterol (LDL-C) were determined by homogeneous method. Serum uric acid was determined by phosphotungstic acid method on automatic biochemical analyzer. All anthropometric measurements and blood biomarkers testing were carried out in accordance with relevant guidelines and regulations.

### Diagnostic standards

According to Health Industry Standards of the People’s Republic of China classifications (12), overweight is defined as a BMI between 24 kg/m2 and 28 kg/m2, while obesity is defined as a BMI greater than 28 kg/m2. Abdominal obesity was defined as waist circumference ≥ 90 cm for men and ≥ 85 cm for women (12). Smoking history was defined as having smoked at least once a day, smoked for more than a year, currently smoking or had quit for less than three years. A history of alcohol consumption was defined as drinking at least once a week, drinking for more than a year, and currently drinking or being sober for less than three years. Hypertension was defined as the self-reported history of hypertension or systolic blood pressure ≥ 140 mmHg and (or) diastolic blood pressure ≥ 90 mmHg (13). Diabetes was defined as the self-reported history of diabetes or fasting blood glucose ≥7.0 mmol/L and (or) OGTT 2-h post-load glucose ≥11.1 mmol/L (14). In our study, we analyzed physical intensity at work and leisure-time exercise. Referring to the Handbook of Cardiovascular Epidemiological Investigation Methods (15), we classify physical intensity at work as (1) very light: sitting (2) light: standing or walking back and forth, driving, housework (3) moderate: general indoor and outdoor activities, yard operation, carrying or lifting light objects, etc. (4) high: heavy industry or agriculture, outdoor construction, field construction, carrying or lifting heavy objects, strenuous sports activities, etc. We classified leisure-time exercise as yes or no, i.e., at least once a week. This definition is relatively simple and is a limitation of our study. In future studies, we will further subdivide the exercise situation based on the WHO definition by frequency and time.

### Statistical analysis

Absolute number (percentage, %) was used to describe the categorical data, and a Chi-Square test was used to compare the difference between different groups. The quantitative data subject to or approximately subject to normal distribution were described by mean ± SD, and the differences between different groups were compared by T-test or analysis of variance. Chi-square trend test was used for the trend analysis of the rate. The multivariable analyses of obesity were conducted by using the non-conditional logistic regression model, and variable screening was conducted by using the forward stepwise selection method. The likelihood ratio (LR), and OR value and its 95% confidence interval were calculated. All statistical analyses were performed with SPSS 23.0 software, and *p* < 0.05 was statistically significant.

## Results

### Demographic and clinical characteristics of the study participants

Among the 10,981 participants (Table 1), 3,881 were men and 7,100 were women. The mean age was 55.07 ± 10.94 years, and men had a higher average age. Men had higher prevalence of marriage, education level above high school degree, smoking, drinking, physical exercise intensity, systolic blood pressure, diastolic blood pressure, and WC (all *p* < 0.001). Women had higher BMI and 2hPG and were more likely to engage in leisure-time physical activity (*p* < 0.05). There was no significant difference in FPG.

**Table 1:** Basic characteristics of the study participants.

Variable	Overall (*n* = 10,981)	Men (*n* = 3,881)	Women (*n* = 7,100)	*p* values
Age (years)	55.07 ± 10.94	56.36 ± 11.18	54.37 ± 10.74	< 0.001
Married (%)	9,990 (91.0)	3,688 (95.0)	6,302 (88.8)	< 0.001
High school education and above (%)	2,614 (23.8)	1,259 (32.4)	1,355 (19.1)	< 0.001
Monthly income ≥ 2,000 CNY (%)	2,075 (19.1)	921 (24.1)	1,154 (16.4)	< 0.001
Current smoking status (%)				< 0.001
Current smoking	2,427 (22.1)	2,132 (54.9)	295 (4.2)	
Never smoking	8,269 (75.3)	1,500 (38.6)	6,769 (95.3)	
Quitting smoking	285 (2.6)	249 (6.4)	36 (0.5)	
Current drinking status (%)				< 0.001
Current drinking	1,874 (17.1)	1,603 (41.3)	271 (3.8)	
Never drinking	8,943 (81.4)	2,155 (55.5)	6,788 (95.6)	
Quitting drinking	164 (1.5)	123 (3.2)	41 (0.6)	
Fatting Food liking (%)				< 0.001
Prefer to eating fatting food	1,816 (16.6)	912 (23.7)	904 (12.8)	
Medium taste	7,356 (67.4)	2,533 (65.8)	4,823 (68.2)	
Prefer to eating light food	1,747 (16.0)	405 (10.5)	1,342 (19.0)	
Physical activity at work (%)				< 0.001
Very light	1,257 (11.4)	541 (13.9)	716 (10.1)	
Light	6,975 (63.5)	1,958 (50.5)	5,017 (70.7)	
Moderate	2,522 (23.0)	1,188 (30.6)	1,334 (18.8)	
High	227 (2.1)	194 (5.0)	33 (0.5)	
Leisure-time physical activity (%)				0.002
No	4,514 (40.1)	1,670 (43.0)	2,844 (40.1)	
Yes	6,467 (58.9)	2,211 (57.0)	4,256 (59.9)	
SBP (mmHg)	130.89 ± 21.32	132.76 ± 20.08	129.87 ± 21.91	< 0.001
DBP (mmHg)	78.50 ± 11.40	80.39 ± 11.18	77.47 ± 11.40	< 0.001
FPG (mmol/L)	5.65 ± 1.79	5.68 ± 1.88	5.63 ± 1.74	0.219
2hPG (mmol/L)	7.91 ± 3.83	7.73 ± 3.82	8.01 ± 3.83	< 0.001
BMI (kg/m^2^)	23.88 ± 3.43	23.60 ± 3.23	24.04 ± 3.52	< 0.001
WC (cm)	81.13 ± 10.26	82.70 ± 9.84	80.27 ± 10.38	< 0.001

Values are presented as mean ± standard deviation (SD), *n* (%); SBP, systolic blood pressure; DBP, diastolic blood pressure; FBG, fasting plasma glucose; 2hPG, 2-h plasma glucose; BMI, body mass index; WC, waist circumference.

### Relationship between BMI and abdominal obesity

The prevalence of abdominal obesity increased with BMI (Table 2). The prevalence of abdominal obesity in individuals with a normal BMI was 8.7%. Of note, 0.5% individuals with a BMI less than 18.5 were still abdominal obesity while 11% of those with a BMI greater than 28 were not.

**Table 2:** The relationship between BMI and abdominal obesity.

BMI	Abdominal obesity		
	Overall (*n* = 10,981)	Men (*n* = 3,881)	Women (*n* = 7,100)
<18.5	2 (0.5)	2 (1.1)	0 (0)
18.5–23.9	482 (8.7)	124 (6.1)	358 (10.3)
24.0–27.9	1,816 (47.6)	553 (41.1)	1,263 (51.1)
≥28.0	1,072 (89.0)	283 (87.3)	789 (89.6)

Values are presented as *n* (%).

### Prevalence of abdominal obesity

Table 3 shows the prevalence of abdominal obesity in different populations. The overall prevalence of abdominal obesity was 30.7%(3372/10981). The prevalence of abdominal obesity in women was higher than that in men (33.9% vs. 24.8%, *p* < 0.001). The overall prevalence of abdominal obesity increased with age, but the prevalence was lowest in men aged 45–54 years, while the prevalence was positively correlated with age in women. The overall prevalence of abdominal obesity in married people was lower than that in single, divorced or widowed people (*p* = 0.032). Whereas the prevalence was higher among married men (25.1% vs. 18.7%, *p* = 0.043) and lower among married women (33.5% vs. 37.3%, *p* = 0.031). The overall prevalence was lower among the highly educated, among women and no statistically significant difference among men. The prevalence of abdominal obesity was higher in people engaged in light physical labor and lower in people engaged in moderate to high intensity physical labor. The prevalence of abdominal obesity in hypertensive and diabetic patients was significantly increased.

**Table 3:** Prevalence of abdominal obesity among participants.

Variable	Overall (*n* = 10,981)	Men (*n* = 3,881)	Women (*n* = 7,100)
All participants (%)	3,372 (30.7)	962 (24.8)†	2,410 (33.9)
Age (years, %)			
35–44	573 (23.4)	184 (23.1)	389 (23.6)
45–54	665 (24.2)	162 (20.4)	503 (25.8)
55–64	1,203 (34.3)	311 (24.0)	892 (40.2)
≥ 65	931 (40.9)	305 (30.7)	626 (48.8)
*p* value	< 0.001	< 0.001	< 0.001
Marital status (%)			
Married	3,038 (30.4)	926 (25.1)	2,112 (33.5)
Widowed, single, and divorced	334 (33.7)	36 (18.7)	298 (37.3)
*p* value	0.032	0.043	0.031
High school education and above (%)			
Yes	572 (21.9)	308 (24.5)	264 (19.5)
No	2,800 (33.5)	654 (24.9)	2,146 (37.4)
*p* value	< 0.001	0.746	< 0.001
Current smoking status (%)			
Current smoking	575 (23.7)	463 (21.7)	112 (38.0)
Never smoking	2,695 (32.6)	412 (27.5)	2,283 (33.7)
Quitting smoking	102 (35.8)	87 (34.9)	15 (41.7)
*p* value	< 0.001	< 0.001	0.199
Current drinking status (%)			
Current drinking	534 (28.5)	395 (24.6)	139 (51.3)
Never drinking	2,780 (31.1)	531 (24.6)	2,249 (33.1)
Quitting drinking	58 (35.4)	36 (29.3)	22 (53.7)
*p* value	0.037	0.505	< 0.001
Physical activity at work (%)			
Very light	374 (29.8)	150 (27.7)	224 (31.3)
Light	2,408 (34.5)	553 (28.2)	1,855 (37.0)
Moderate	551 (21.8)	223 (18.8)	328 (24.6)
High	39 (17.2)	36 (18.6)	3 (9.1)
*p* value	< 0.001	< 0.001	< 0.001
Leisure-time physical activity (%)			
No	1,236 (27.4)	353 (21.1)	883 (31.0)
Yes	2,136 (33.0)	609 (27.5)	1,527 (35.9)
*p* value	< 0.001	< 0.001	< 0.001
Hypertension (%)			
Yes	1,754 (42.8)	520 (33.8)	1,234 (48.1)
No	1,618 (23.5)	442 (18.9)	1,176 (25.9)
*p* value	< 0.001	< 0.001	< 0.001
Diabetes mellitus (%)			
Yes	1,023 (45.9)	270 (34.4)	753 (52.1)
No	2,349 (26.8)	692 (22.3)	1,657 (29.3)
*p* value	< 0.001	< 0.001	< 0.001

†Compared with women, *p* < 0.001. The number and prevalence of abdominal obesity among participants were provided.

### Multivariable-adjusted ORs and 95% CI for abdominal obesity

Table 4 shows the results of multivariate logistic regression. Abdominal obesity was positively correlated with female, age, marriage, alcohol consumption, hypertension, and diabetes, but negatively correlated with high school education and above, smoking, moderate and high physical activity at work.

**Table 4:** Multivariable logistic regression analysis of abdominal obesity and the associated factors.

	OR (95%CI)
Sex	
Men	1.000 (reference)
Women	1.575 (1.389–1.785)
Age (years)	
35–44	1.000 (reference)
45–54	0.980 (0.859–1.119)
55–64	1.277 (1.128–1.447)
≥ 65	1.463 (1.270–1.686)
Married	1.221 (1.051–1.418)
High school education and above	0.648 (0.579–0.724)
Current smoking status	
Current smoking	1.000 (reference)
Never smoking	1.292 (1.118–1.494)
Quitting smoking	1.495 (1.127–1.984)
Current drinking status	
Current drinking	1.000 (reference)
Never drinking	0.695 (0.603–0.802)
Quitting drinking	0.766 (0.530–1.105)
Physical activity at work	
Very light	1.000 (reference)
Light	1.076 (0.937–1.235)
Moderate	0.766 (0.651–0.901)
High	0.706 (0.482–1.033)
Hypertension	1.926 (1.760–2.109)
Diabetes mellitus	1.719 (1.552–1.904)

Values are presented as odds ratios (95% confidence interval).

## Discussion

This study assessed the prevalence and related factors of abdominal obesity in adults aged 35–79 years in Chengdu and Chongqing from September 2013 to March 2014. Overall, the prevalence of abdominal obesity was 30.7%. Previous data on the prevalence of abdominal obesity in the region was scant. A previous survey of 1,061 middle-aged and older adults in Chengdu suggested that the prevalence of abdominal obesity increased significantly (23.7% vs. 50%) 15 years ago from 1992 to 2007 (16). However, in this study, waist circumference ≥ 85 cm for men and waist circumference ≥ 80 cm for women were selected as the diagnostic criteria for abdominal obesity, so the prevalence was relatively high. It has to be mentioned that many years ago, the definition of abdominal obesity among middle-aged and older adults in urban communities in Chengdu adopted the 2004 Guidelines for the Prevention and Control of overweight and obesity among adults in China, while the definition of abdominal obesity in this study adopted the Health Industry Standards of the People’s Republic of China classifications in 2013. The comparison of prevalence calculated by different standards is not accurate. In another study, the prevalence of abdominal obesity in 4,551 Han people aged 20–80 years in Guizhou in 2012 was 26.8% (17). However, there is no separate data of urban community population for the prevalence of Han population in Guizhou. Our study to some extent filled the lack of data in this area, which is one of the important reasons for our research. Collectively, abdominal obesity is common among adults in southwest China, and with the rapid economic development of southwest China in recent years, overeating, lack of exercise and other unhealthy lifestyles, the prevalence of abdominal obesity has also increased significantly.

A national study of 441,306 participants aged 18 and older between October 2012 and December 2015 showed a prevalence of 29.1% (6). This study indicates that the prevalence of abdominal obesity in southwest China is close to the national level. Compared with other regions in China, abdominal obesity has obvious regional differences (18–20). Overall, the prevalence of abdominal obesity is higher in the North than in the South, which may be due to the cold climate in the North and the difference in eating habits between the north and the South (21, 22). The prevalence of abdominal obesity in Beijing and Guangzhou was higher than that in surrounding areas (6, 23), and the prevalence of abdominal obesity has increased with economic development in recent years, indicating that abdominal obesity may also be related to economic development.

This study further analyzed the factors associated with abdominal obesity, and the results showed that women, age, marriage, alcohol consumption, hypertension and diabetes were positively correlated with abdominal obesity, consistent with previous studies (6, 19, 20). However, the education level above high school, smoking, moderate and heavy physical labor were negatively correlated with abdominal obesity. The prevalence of abdominal obesity in females was higher than that in males (33.9% vs. 24.8%, *p* < 0.001), and this difference increases from the age group over 45 years. Possible explanation is that middle-aged and older women are less concerned with body image management than men, such as waist circumference, and changes in estrogen levels, particularly after menopause (24), also contribute to this difference. For the gender difference in the prevalence of abdominal obesity in different marital status, married men are more likely to be abdominal obesity, while this situation is opposite in women. The possible reason is that the diet status of unmarried men in most cases is often simple, that is, dietary intake is low. After marriage, men tend to eat more because their wives usually prepare rich meals for their families. In addition, after marriage, men’s work pressure and life pressure become greater, which affects the hormone levels of men, may lead to weight gain. For women, after marriage, in addition to working, they also need to complete housework, take care of children, and take care of their spouse, etc., and their physical activity increases significantly, which may lead to weight loss and a decrease in waist circumference. Several factors may contribute to obesity in married women but may be largely offset by increased physical activity. In this study, abdominal obesity was more common in people over 55 years of age, possibly due to decreased physical activity, decreased limb muscle mass, and changes in postmenopausal hormone levels (25). The older adults are at high risk of cardiovascular diseases. In addition to the prevention and treatment of hypertension and glycolipid abnormalities, the prevention and management of abdominal obesity are also very important. As a Chinese saying goes a thin body for an old man is beyond all money value. Drinking alcohol is quite common in China. While it has been widely recognized that alcohol consumption increases the risk of obesity, previous studies have reported inconsistent results on the relationship between alcohol consumption and obesity (26, 27). A study of abdominal obesity and drinking patterns in normal-weight middle-aged adults in South Korea found that in normal-weight middle-aged adults, the amount of alcohol consumed per drink affected abdominal obesity (28). The possible explanation is that high alcohol intake increases energy intake, leading to fat accumulation, and the endocrine changes caused by alcohol intake seem to be important (26). Alcohol alters steroid metabolism in the liver, leading to fat accumulation (29). Reducing alcohol consumption may help reduce the prevalence of abdominal obesity, thereby reducing cardiovascular disease, metabolic syndrome and other related diseases. In addition, our study showed that hypertension and diabetes were positively correlated with the prevalence of abdominal obesity. Previous studies have discussed the important impact of obesity on cardiovascular disease (30, 31). So we should actively control these underlying diseases that are associated with obesity. Interestingly, the prevalence of abdominal obesity was lower in people with higher levels of education, consistent with previous national studies (6). People with higher levels of education usually have more stable jobs and incomes and a higher standard of living. This could be due to greater health awareness, a better diet, as well as a more regular lifestyle and physical activity. In our study, current smokers were associated with a reduced prevalence of abdominal obesity, while former smokers were associated with an increased prevalence of abdominal obesity. The results are consistent with previous studies (6, 32), including a national study of a Chinese population (6). But the link between smoking and abdominal obesity remains controversial. Many previous studies (33–37) have observed an increased prevalence of abdominal obesity in people who smoke, such as adolescents (33, 34), women (35, 36), people with type 2 diabetes (37), heavy smokers (37), and former smokers (38). The possible mechanisms in the association between smoking and abdominal obesity are as follows. Some studies have suggested that nicotine causes insulin resistance (39, 40) and increases cortisol levels (41), factors that may be linked to belly fat accumulation. In addition, smokers are more likely to have unhealthy lifestyle habits, such as increased alcohol intake and lack of physical activity, which may contribute to abdominal obesity (42). Smokers gain weight after quitting, which may contribute to abdominal obesity in people who give up smoking (43, 44). Female smokers have a higher prevalence of abdominal obesity, and this sex difference may be explained by the anti-estrogen effects of nicotine (45). Results on the prevalence of abdominal obesity in current smokers are inconsistent (6, 32, 35–37). Smokers are more likely to have cardiovascular disease, diabetes, and metabolic syndrome (46, 47), although they may have a lower BMI than nonsmokers (48). So smoking is not recommended as a way to control weight.

The current situation of abdominal obesity in southwest China is not optimistic, which will further lead to diabetes and cardiovascular diseases, and thus could contribute to heavier medical burden. As is demonstrated in this study, more preventive measures should be taken to reduce the prevalence of abdominal obesity. For example, a healthy lifestyle, lower alcohol consumption, strict control of blood pressure and blood glucose, and active participation in physical exercise are of great significance for controlling abdominal obesity.

This study has several limitations. In this study, approximately 18% of participants were excluded due to the lack of important information, such as demographic information, anthropometry, and obesity measurements, which could lead to potential selection bias. This study selected five communities in Chengdu and Chongqing, the most developed cities in southwest China. However, these communities may not fully represent the urban areas in southwest China. This study is a cardiovascular metabolism study carried out in urban communities in southwest China. The original target population of this study is middle-aged and older adults, mainly including people over 40-years-old, but also some people between 35 and 40 years old. People aged 18–34 were not included. This is also a limitation of this study. Finally, other alternative measures of obesity such as waist-to-height ratio are of great significance. In future study, we expect to make a comprehensive comparison between these measures of obesity (e.g., waist circumference, waist-to-hip circumference, waist-to-height ratio) and yield a more accurate indicator for abdominal obesity.

Our study confirms the prevalence of abdominal obesity in southwest China is approximate to the national level. The prevalence of abdominal obesity was positively correlated with age, sex, marriage, alcohol consumption, hypertension and diabetes, and negatively correlated with high education level, smoking and Physical activity. The current situation of abdominal obesity in southwest China is not optimistic, our study provides evidence for the active prevention and control of abdominal obesity and the promotion of public health. Last but not least, this study collected socioeconomic and lifestyle variables such as monthly household income and eating habits of the research subjects in the last year, which were not reported in this study because the results were not significant. These variables have been reported with the results of previous studies on hypertension and diabetes (49–51). Otherwise, this study also has certain limitations, the cross-sectional nature of the survey cannot show a causal relationship between relevant influencing factors and the prevalence of abdominal obesity. In addition, the potential for recall bias or self-reporting errors in data collection may bring some bias to the study results.

## Data availability statement

The original contributions presented in the study are included in the article/supplementary material, further inquiries can be directed to the corresponding authors.

## Ethics statement

The studies involving human participants were reviewed and approved by the Ethics Committee of Chengdu Second People's Hospital (no 2013015). The patients/participants provided their written informed consent to participate in this study.

## Author contributions

CH and X-bH conceived and designed the study. CH, YZ, W-WT, J-XL, Y-MH, and YL analysed the data, drafted, and revised the manuscript. W-WT, T-DW, and X-bH advised on the interpretation of results and were responsible for the research. All authors contributed to the article and approved the submitted version.
